# Supplementary material for: A DFT Approach to the Surface-Enhanced Raman Scattering of 4-Cyanopyridine Adsorbed on Silver Nanoparticles
Source: Nanomaterials (Basel). 2019 Aug 28;9(9):1211. doi: 10.3390/nano9091211 (PMC6780094; doi:10.3390/nano9091211)
Supplement: Supplementary file 1 [file nanomaterials-09-01211-s001.pdf]

nanomaterials

Supplementary Materials

A DFT Approach to the Surface-Enhanced Raman Scattering of 4-Cyanopyridine Adsorbed on Silver Nanoparticles.

Isabel López-Tocón [1,*], Samuel Valdivia [1], Juan Soto [1], Juan Carlos Otero [1], Francesco Muniz-Miranda [2,#], Maria Cristina Menziani [2], and Maurizio Muniz-Miranda [3]

1 Andalucía Tech, Unidad Asociada IEM-CSIC, Departamento de Química Física, Facultad de Ciencias, Universidad de Málaga, Málaga, Spain; tocon@uma.es, svaldivia@uma.es, soto@uma.es, jc_otero@uma.es
2 Department of Chemical and Geological Sciences, University of Modena and Reggio Emilia, Via Campi 103, 41125 Modena, Italy; f.muniz-miranda@chimieparistech.psl.eu; mariacristina.menziani@unimore.it
3 Department of Chemistry "Ugo Schiff", University of Florence, Via della Lastruccia 3, 50019 Sesto Fiorentino, Italy ; maurizio.muniz@unifi.it
# present address: Chimie ParisTech, PSL Research University, CNRS, Institute of Chemistry for Life and Health Sciences, F-75005 Paris, France
* Correspondence: tocon@uma.es

Received: 24 July 2019; Accepted: 25 August 2019; Published: 28 August 2019

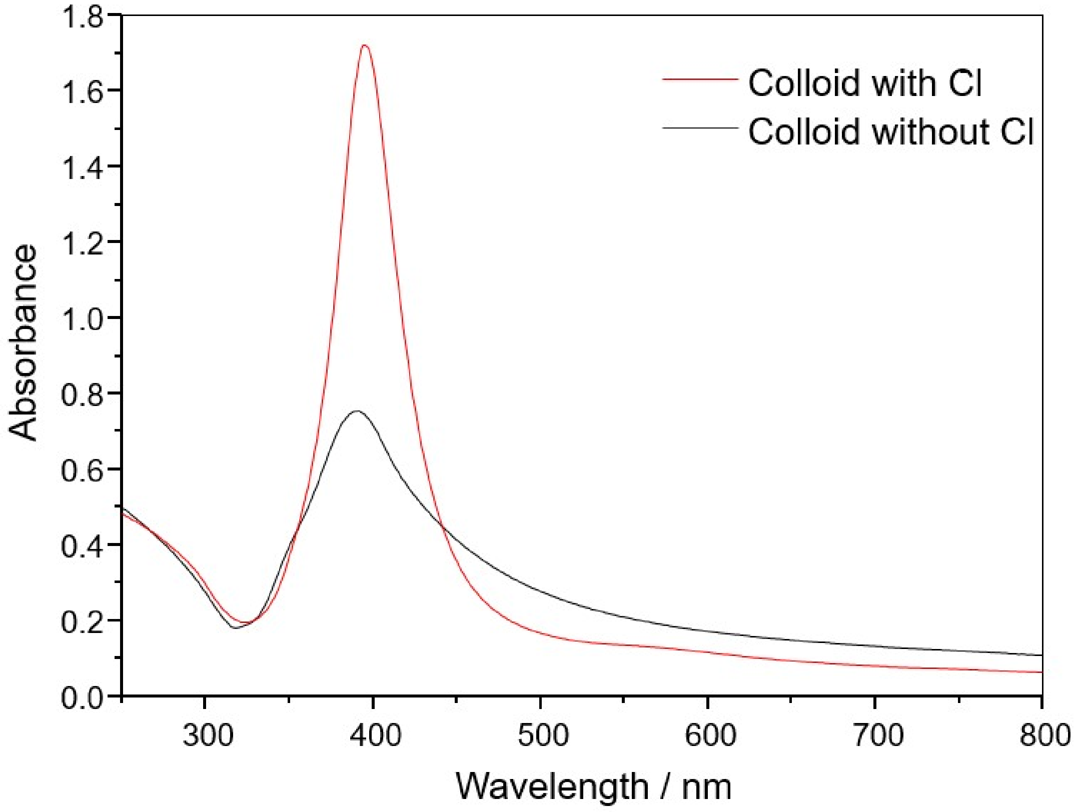

Figure S1. UV-visible extinction spectra of borohydride silver colloid used as SERS substrate before and after addition of chloride ions.

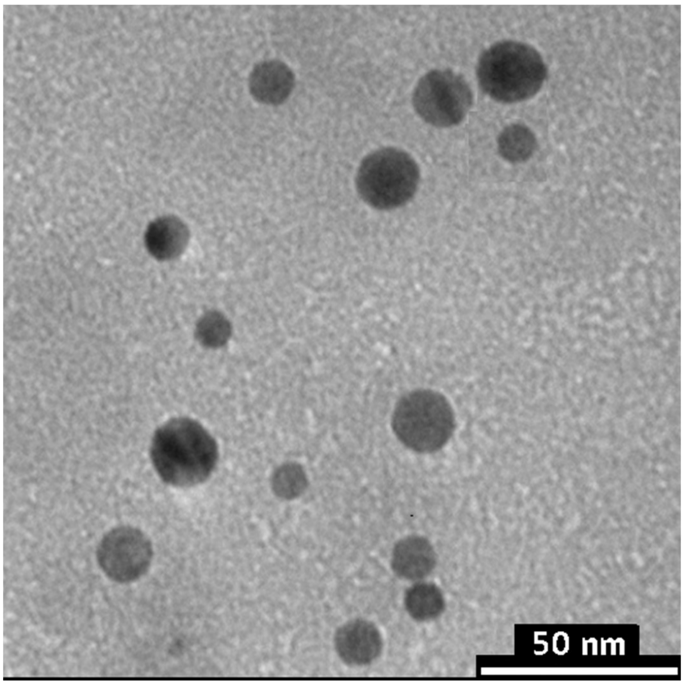

Figure S2. TEM image of Ag/Cl⁻nanoparticles.

Table S1. Experimental Raman and SERS wavenumbers (cm^{-1}) of 4-cyanopyridine (4CNPy) and those calculated B3LYP/LanL2DZ in its ground electronic state $S_0;{}^1A_1$. A vibrational assignment of 4CNPy is proposed and correlated with that of pyridine.

Experimental Pyridine[a]				B3LYP/ LanL2DZ	Experimental 4CNPy			
**Liquid**	**Solution 1M**	**Symm.**	**Assignment[b]**	**4CNPy**	**Raman[c] Solid**	**Raman[c] Solution**	**SERS[c] without Cl^- ions**	**SERS[c] with Cl^- ions**
3061	3077	A_1	2,ν(CH)	3248	3070 (vw)	3084 (w)	3088 (vw)	
3065			20a,ν(CH)	3218	3033 (vw)			
			ν(CN)	2271	2248 (vs)	2250 (vs)	2248 (s)	2248 (s)
1582	1596		8a,ν_{ring}	1623	1597 (s)	1602 (s)	1608 (s)	1608 (s)
1484	1492		19a,δ(CH)	1507	1498 (vw)	1498 (vw)	1510 (m)	1507 (vw)
1217	1221		9a,ν_{ring}	1254	1240 (m)	1250 (vw)	1220 (s)	1221 (vw)
			ν(CX),ν_{ring}+ν(CN)	1230	1193 (s)	1198 (s)	1202 (s)	1196 (s)
1069	1071		18a,δ(CH)	1092	1075 (m)	1072 (vw)		1072 (m)
1033	1037		12;δ_{ring}	987	991 (vs)	1004 (s)	1010 (s)	1012 (s)
993	1005		1,ν_{ring}+ν(CN)	774	775 (m)	784 (m)	790 (w)	790 (w)
604	618		6a,δ_{ring}	460	454 (s)	468 (m)		472 (s)
3079		B_2	20b,ν(CH)	3243	3070 (vw)			
			7b,ν(CH)	3216	3033 (vw)			
1574	1578		8b,ν_{ring}	1584	1548 (vw)	1556 (vw)		
1438			19b,δ(CH)	1440			1410 (m)	1412 (w)
1358			3,δ(CH)	1369	1341 (w)	1330 (vw)	1334 (m)	1336 (w)
1230	1231		14,ν_{ring}+δ(CCN)	1290				
1149	1153		15,δ_{ring}	1116	1113 (vw)			
654	654		6b,δ_{ring}	684	668 (s)	672 (m)	676 (w)	672 (w)
			δ(CCN)	563	560 (w)	566 (w)		562 (w)
			CCC,CN skeletal	169	189 (s)			
983		A_2	17a,γ(CH)	1022	1036 (vw)			
882			10a,γ(CH)	919				
377			16a,τ_{ring}	386	372 (w)	378 (vw)		384 (vw)
942		B_1	10b,γ(CH)	1015	1000 (w)			
710			17b,γ(CH)	869	890 (vw)	900 (vw)		
752			4,τ_{ring}	768	747 (vw)	754 (vw)		
			γ(CCN)	596	552 (vw)	552 (vw)		552 (vw)
407			γ(CN) + 16b,τ_{ring}	398				394 (vw)
			CCC,CN skeletal	150	147 (w)			

[a]Reference [46] [b]Wilson's nomenclature from Varsanyi, G. *Vibrational spectra of benzene derivatives*, Academic Press, New York, 1969. ν:stretching, δ:in-plane deformation, γ:out-of-plane deformation and τ:torsion. [c] vs: very strong, s: strong, m: medium, w: weak, vw: very weak.

Table S2. B3LYP/LanL2DZ optimized geometries of 4CNPy and Ag-4CNPy surface complexes, in the ground electronic state $S_0;{}^1A_1$.

	**4CNPy**	**Ag-4CNPy**			
		**Ag-CN**		**Ag-N**	
**Structure**	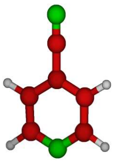	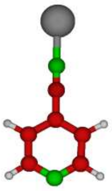	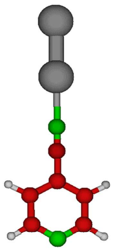	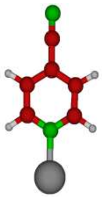	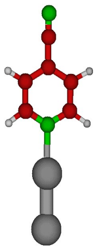
**Parameters**[a]		**Ag^+-CN**	**Ag_2-CN**	**Ag^+-N**	**Ag_2-N**
$R_{1/6}$	1.3578	1.3573	1.3577	1.3671	1.3596
$R_{2/5}$	1.4061	1.4064	1.4062	1.3989	1.4028
$R_{3/4}$	1.4136	1.4163	1.4136	1.4140	1.4133
R_7	1.4407	1.4321	1.4383	1.4373	1.4403
R_8	1.1821	1.1772	1.1758	1.1809	1.1814
$R_{9/12}$	10863	1.0855	1.0860	1.0838	1.0849
$R_{10/11}$	1.0850	1.0851	1.0850	1.0844	1.0846
α_1	117.84	118.66	118.04	119.17	118.79
$\alpha_{2/6}$	123.26	123.03	123.21	121.95	122.49
$\alpha_{3/5}$	118.44	117.72	118.20	119.10	118.69
α_4	118.75	119.82	119.12	118.71	118.83
$\beta_{1/4}$	116.03	116.15	116.08	117.51	116.46
$\beta_{2/3}$	120.69	120.56	120.69	119.93	120.36
β_5	120.62	120.09	120.44	120.64	120.58
**Ag-CN**		2.1572	2.4160		
**Ag-N**				2.1848	2.3512

[a]Bond lengths in Angstroms and angles in Degrees. See Figure below for symbols.

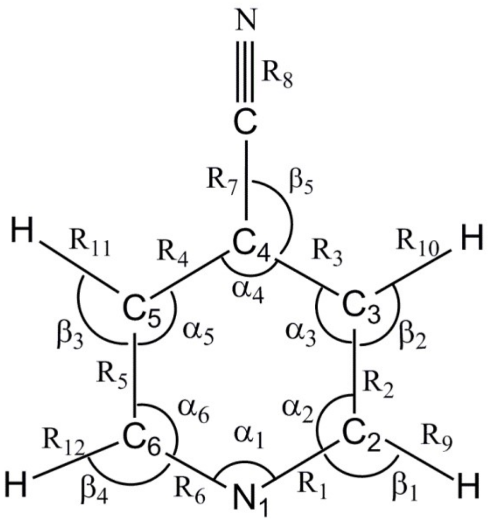

Table S3. B3LYP/LanL2DZ wavenumbers of 4CNPy and the Ag-4CNPy surface complexes, in the ground electronic state S_0;$^1A_1$.

		Wavenumbers / cm^{-1}				
**Symm.**	**Assignment**[a]	**4CNPy**	**Ag^+-N**	**Ag^+-CN**	**Ag_2-N**	**Ag_2-CN**
**A_1**	2,ν(CH)	3248	3266	3253	3255	3249
	20a,ν(CH)	3218	3246	3231	3223	3222
	ν(CN)	2271	2284	2297	2277	2297
	8a,ν_{ring}	1623	1651	1603	1638	1620
	19a,δ(CH)	1507	1524	1501	1515	1506
	9a,ν_{ring}	1254	12665	1256	1254	1255
	ν(CX),ν_{ring}+ν(CN)	1230	1240	1228	1233	1231
	18a,δ(CH)	1092	1085	1089	1089	1092
	12;δ_{ring}	987	1027	983	1010	986
	1,ν_{ring}+ν(CN)	774	797	782	786	778
	6a,δ_{ring}	460	493	497	476	475
**B_2**	20b,ν(CH)	3243	3262	3248	3251	3245
	7b,ν(CH)	3216	3245	3230	3231	3220
	8b,ν_{ring}	1584	1574	1576	1582	1584
	19b,δ(CH)	1440	1457	1439	1446	1440
	3,δ(CH)	1369	1377	1370	1370	1370
	14,ν_{ring}+δ(CCN)	1290	1304	1289	1295	1289
	15,δ_{ring}	1116	1136	1119	1125	1117
	6b,δ_{ring}	684	680	680	683	684
	δ(CCN)	563	560	583	562	575
	CCC,CN skeletal	169	190	195	180	200
**A_2**	17a,γ(CH)	1022	1027	1024	1024	1023
	10a,γ(CH)	919	912	909	916	917
	16a,τ_{ring}	386	401	372	391	382
**B_1**	10b,γ(CH)	1015	1021	1016	1017	1015
	17b,γ(CH)	869	884	861	874	865
	4,τ_{ring}	768	761	758	766	966
	γ(CCN)	596	592	598	595	596
	γ(CN) + 16b,τ_{ring}	398	396	409	402	403
	CCC,CN skeletal	150	176	165	170	167

[a]Wilson's nomenclature from Varsanyi, G. *Vibrational spectra of benzene derivatives*, Academic Press, New York, 1969. ν:stretching, δ:in-plane deformation, γ:out-of-plane deformation and τ:torsion.

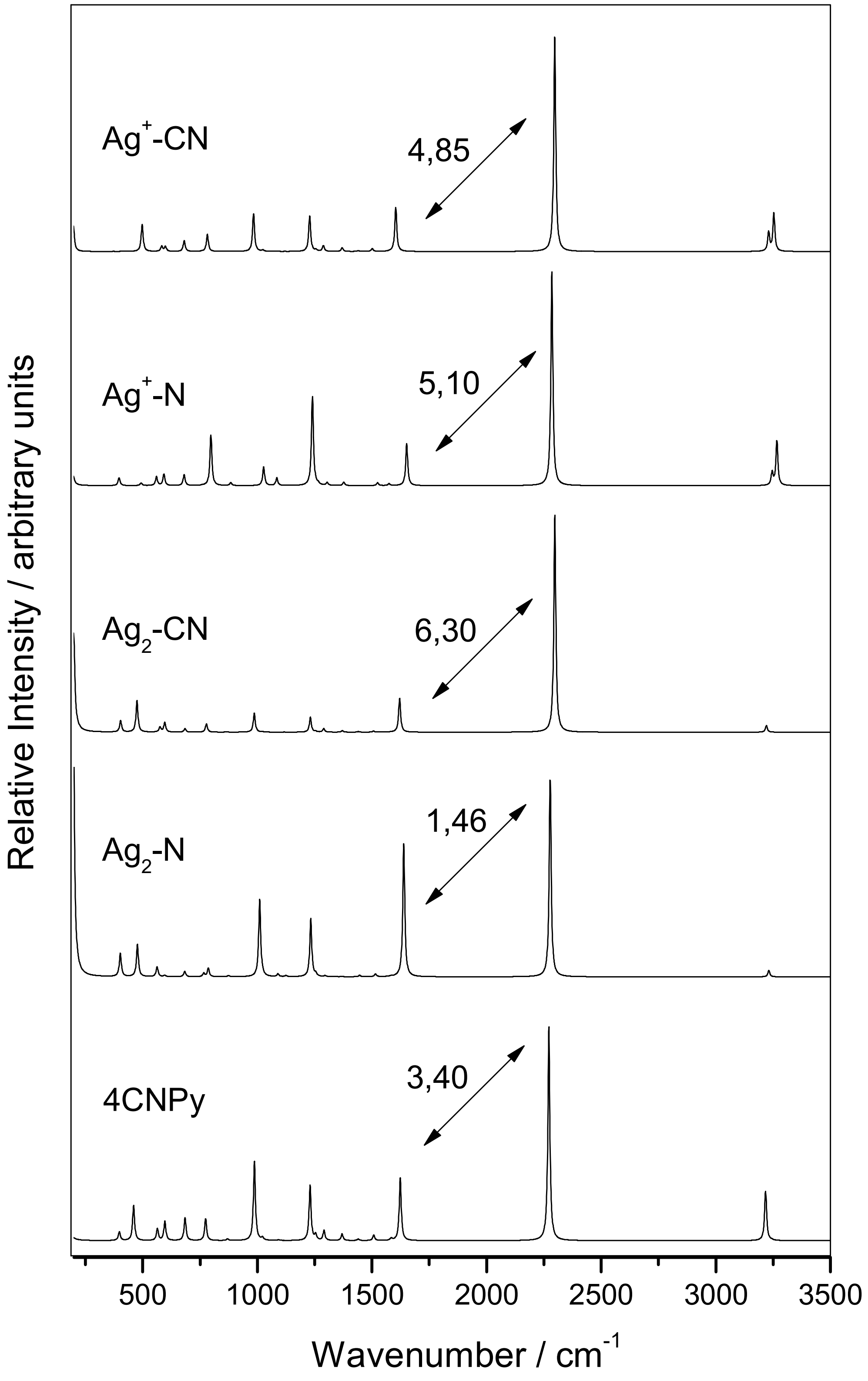

Figure S3. B3LYP/LanL2DZ Raman spectra of 4CNPy isolated and its different complexes in the whole wavenumber range. The intensity ratio between CN (2250 cm^{-1}) and 8a (1600 cm^{-1}) bands are indicated.

Table S4. B3LYP/LanL2DZ Mulliken´s charges of 4CNPy and the Ag-4CNPy surface complexes, in the ground electronic state S_0;$^1A_1$.

	Mulliken Charges / a.u.				
**Assignment[a]**	**4CNPy**	**Ag^+-N**	**Ag^+-CN**	**Ag_2-N**	**Ag_2-CN**
N_1	-0.008	-0.286	0.025	-0.154	0.002
$C_{2,6}$	-0.293	-0.119	-0.278	-0.188	-0.299
$C_{3,5}$	-0.288	-0.269	-0.265	-0.281	-0.274
C_4	0.406	0.419	0.395	0.397	0.400
$H_{9,12}$	0.247	0.264	0.274	0.263	0.253
$H_{10,11}$	0.254	0.288	0.264	0.264	0.258
C	-0.228	-0.217	-0.001	-0.238	-0.185
N	-0.009	0.065	-0.167	0.009	0.067
Ag, Ag_2		0.69	0.75	-0.14	-0.15
ΔQ[b]		-0.31	-0.25	-0.14	-0.15

[a]See Table S2 for definition of the atoms. [b]Charge difference between that of the complex and the silver atoms.

© 2019 by the authors. Licensee MDPI, Basel, Switzerland. This article is an open access article distributed under the terms and conditions of the Creative Commons Attribution (CC BY) license (http://creativecommons.org/licenses/by/4.0/).
